# Integrating 3Rs approaches in WHO guidelines for the batch release testing of biologicals: Responses from a survey of National Control Laboratories and National Regulatory Authorities

**DOI:** 10.1016/j.biologicals.2023.101721

**Published:** 2023-11

**Authors:** Elliot Lilley, Martijn Bruysters, Pradip Das, Simeon Gill, Richard Isbrucker, David Jones, Anthony Holmes

**Affiliations:** a NC3Rs, London, UK; b National Institute of Public Health and the Environment, Bilthoven, the Netherlands; c Biological E Limited, Hyderabad, India; d AstraZeneca, Cambridge, UK; e World Health Organisation, Geneva, Switzerland; f Consultant - formally MHRA, UK

**Keywords:** 3Rs, Batch release testing, Quality control, Vaccines, Biological therapeutics, WHO, NC3Rs, Non-animal testing strategies, Survey, Vaccine manufacturers, National Control Laboratories, National Regulatory Authorities

## Abstract

The UK National Centre for the Replacement, Refinement, and Reduction of Animals in Research (NC3Rs) is reviewing World Health Organization (WHO) manuals, guidelines and recommendations for vaccines and biotherapeutics to identify the extent to which animal-based testing methods are described. The aim is to recommend where updates to these documents can lead to an increased and more harmonised adoption of 3Rs principles (i.e. Replacement, Reduction and Refinement of animal tests) in the quality control and batch release testing requirements for vaccines and biotherapeutics. Improved adoption of 3Rs principles and non-animal testing strategies will help to reduce the delays and costs associated with product release testing. Developing recommendations that are widely applicable by both the manufacturers and national regulatory authorities for vaccines and biological therapeutics globally requires a detailed understanding of how different organisations view the opportunities and barriers to better integration of the 3Rs. To facilitate this, we developed and distributed a survey aimed at individuals who work for national regulatory authorities (NRAs) and/or national control laboratories (NCLs). In this paper, we present the key findings from this survey and how these will help inform the recommendations for wider integration of 3Rs approaches by WHO in their guidance documents applicable to the quality control and batch release testing of vaccines and biotherapeutics.

## 1 Introduction

Vaccines and biological therapeutics are important for human health. They are derived from biological sources and are inherently heterogeneous, resulting in potential variations of the same product from batch to batch, so it is essential that the quality of these products is adequately controlled. Batch to batch consistency within a product quality profile is maintained through adequate control of the manufacturing process. The combination of adequate manufacturing process control and additional routine monitoring where appropriate during manufacture is a strategy that sufficiently maintains assurance of product safety and efficacy throughout its entire lifecycle. The World Health Organization (WHO) has established international norms and standards for this purpose and their guidelines and recommendations carry significant influence, being adopted extensively throughout governmental regulatory networks globally. The UK National Centre for the Replacement, Refinement, and Reduction of Animals in Research (NC3Rs) has undertaken a review of animal-based testing methods used for quality control purposes described in WHO written standards for vaccines and biological therapeutics. The aim is to identify opportunities for increased and more harmonised adoption of 3Rs principles (i.e. Replacement, Reduction, and Refinement of animal tests) to accelerate the adoption of the most scientifically relevant testing methods available, to reduce animal use and to reduce the delays and costs associated with product release testing [1].

Significant challenges and opportunities exist that affect the uptake of 3Rs methods. Understanding these from the perspectives of the relevant communities is essential in developing recommendations that will facilitate the widest adoption of quality control and batch release testing approaches capable of reducing animal use. The two key stakeholder groups in this effort are the manufacturers of vaccines and biological therapeutics and the competent authorities responsible for regulation of safe and efficacious medicinal products in their territory. To understand their perspective on this issue the NC3Rs has surveyed both groups. In this paper, the key findings from the regulators survey are presented. Manufacturer perspectives are described in a separate publication [2].

## 2 Survey information (method)

The survey was distributed as a Microsoft Excel™ file and consisted of three sections to collect:

1. Demographic data,
2. Responses from National Regulatory Authorities (NRAs): regarding current practices with respect to how local, national or international rules govern how quality control and batch release testing of vaccines and biological therapeutics is regulated. The survey further explores opportunities and barriers to the adoption of 3Rs and non-animal methods,
3. Responses from National Control Laboratories (NCLs): regarding current practices with respect to the use of animal-based methods for quality control and batch release testing of vaccines and biological therapeutics. The survey further explores opportunities and barriers to the adoption of 3Rs and non-animal methods.

Respondents could complete the survey from the perspective of an NRA, an NCL or both.

Prior to distribution, the survey passed ethical review by the Royal Veterinary College (RVC, London, UK) Social Science Research Ethical Review Board (RVC ref: URN: SR2021-0169). It was launched in January 2022 and formally closed in April 2022 (three responses were received after this date and are included in the analysis in this paper). The survey was distributed (via a link to the NC3Rs website) through the WHO NCL network, social media, direct email to networks and industry newsletters and websites. The survey data received was fully anonymised prior to its analysis. All product and animal test data were randomised and any information that could identify individuals or their employers was redacted. The anonymised dataset was shared with an international group of experts in the production, regulation and quality control of biological therapeutics to support data analysis and interpretation and who are co-authors of this paper.

## 3 Survey data (results)

The anonymised data from the survey analysis is available as supplementary materials (file: Supplementary_materials_regulatory_survey_raw_data.xslx)

### 3.1 Survey respondent demographics

Thirty four responses were received with 31 completed surveys (two responses came from NRAs indicating that they did not perform any animal testing and one incomplete response was received). Unfortunately, not all respondents answered all of their respective questions and, in the interests of full disclosure, the number of responses per question is reported for each question in the results section. The nature of the survey dissemination means that we do not know how many individual NRAs and NCLs were aware of the survey and therefore, we cannot judge the overall response rate. The questions from the survey are reproduced in appendix 1.

**Fig. 1:**
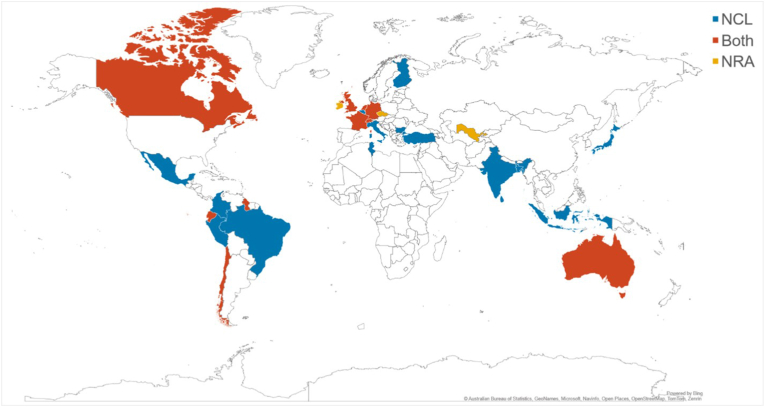
Map illustrating countries that responded to the survey.

Completed surveys were received from 29 different countries (Fig. 1). Two separate responses (NRA and NCL) were received from both the United Kingdom and the Netherlands. Most responses (11/29) were from respondents based in Europe, followed by the Asia-Pacific region (8/29) and the Americas (8/29); with the remainder of the responses from Africa (1/29) and Oceania (1/29). Most responses came from NCLs (18/31), 11 respondents included data from organisations that act as both NRA and NCL in their country. Only two surveys were returned from organisations that act solely as the NRA for their country. In total, there were 13 sets of NRA data and 29 sets of NCL data.

## 4 NRA responses

### 4.1 Understanding the current regulatory requirements for animal testing and use of non-animal technologies (NATs)

In the NRA specific section of the survey, a series of questions were asked about current practices regarding regulation of quality control and batch release testing of vaccines and biological therapeutics. The focus was current regulatory requirements for animal testing and how NATs (assay modalities that do not use animals; e.g*. in vitro* potency assays, use of the monocyte activation test to replace the rabbit pyrogen test) are perceived.

Most (61 %; 8/13) NRAs that responded to the survey indicated that they shared regulatory decisions with other countries within their region with regards to batch control testing. This is encouraging if this regulatory harmonisation results in testing not being repeated by individual member states within a region. When asked if they had discussed the use of NATs with manufacturers only a third of respondents (38 %; 5/13) indicated that this was the case. Of these, three indicated that these discussions occurred at both pre- and post-approval stages whilst one respondent indicated that discussions only occur at the pre-approval stage.

A little over two thirds (69 % 9/13) of NRAs indicated that their regional/national guidance materials related to quality control and batch release testing was aligned to WHO guidelines. Respondents were asked if there were national regulations that mandated animal testing for quality control and batch release testing; 61 % (8/13) NRAs indicate that this was the case.

**Fig. 2:**
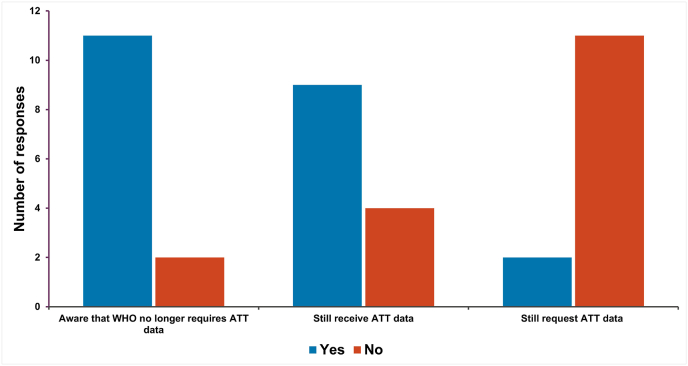
Awareness of the WHO decision to remove the ATT from their requirements and whether NRAs who responded to the survey still receive or request ATT test data.

The Abnormal Toxicity Test (ATT) (also referred to as the General Safety Test (GST) or test for innocuity) has been widely acknowledged as being scientifically questionable, non-reproducible and non-specific [[3], [4], [5]]. The WHO Expert Committee on Biological Standardization (ECBS) followed the United States of America Food and Drug Administration (FDA), the European Pharmacopeia (Ph Eur) and others in removing this test from their requirements in 2018 [6]. Respondents were asked if they were aware that the WHO had removed the requirement to perform this test, whether they receive submissions with ATT test data and whether they request ATT test data. There was good awareness that the WHO had removed this test (85 %; 11/13) and the same proportion of NRAs indicated that they did not request this data. However, despite this, most NRAs who completed the survey (69 %; 9/13) still receive ATT test data (Fig. 2).

This is in alignment with the perspective of biological manufacturers where most (80 % of those responding to our previous survey) indicated that they were aware that the WHO had removed the requirement for this test but, despite this, 57 % still performed the test [2]. This could be because manufacturers may market their products to multiple regions, some of which still require the ATT to satisfy their national regulations/requirements. This survey received 13 responses from NRAs and clearly this is not necessarily representative of all global NRAs, indeed other NRAs may still request ATT data. Lack of global harmonisation with respect to requirements to perform this test is a critical factor in its continued use.

### 4.2 3Rs awareness and practice

A significant barrier to the adoption of 3Rs approaches is a lack of awareness that appropriate NATs are available. We explored if this was also the case for the vaccines and biological therapeutics community to better understand how best to support the adoption of these approaches.

**Fig. 3:**
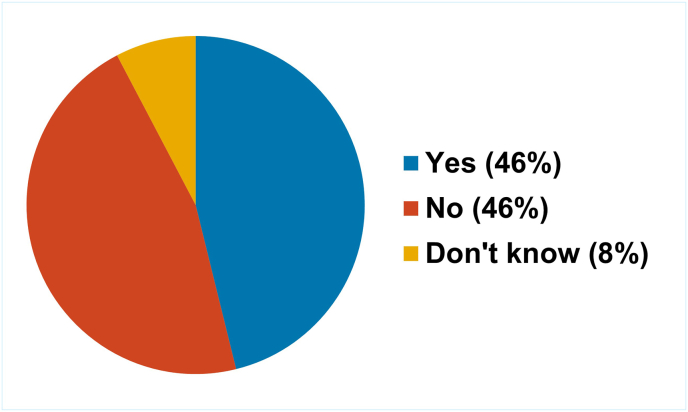
Proportion of NRAs that have a specific 3Rs policy.

Respondents were asked if their own regulatory authority had a specific 3Rs policy (Fig. 3). Responses were mixed (one respondent did not answer the question, one indicated that they did not know); 42 % (6/13) stated that they did have a policy and 50 % (6/13) that they did not. However, all NRAs that responded to the survey indicated that NATs, when available and scientifically appropriate, should be used for quality control and batch release testing.

**Fig. 4:**
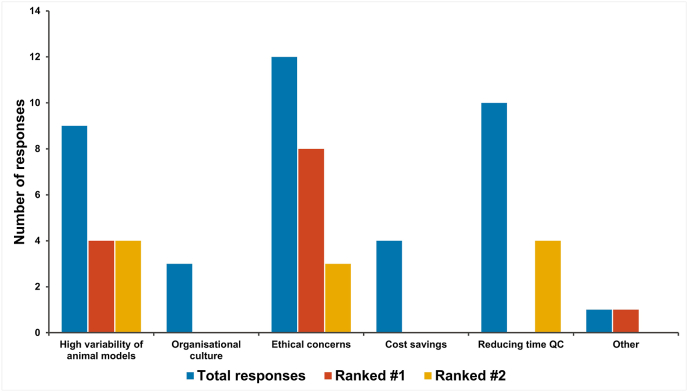
Reasons why NRAs regard adoption of NATs in quality control and batch release testing to be important.

When asked why it was important that NATs were used in this context (respondents were able to choose multiple options and to rank the top two responses), the most frequently chosen options were ethical concerns for animal welfare, reducing time for quality control testing and the high variability of data generated in animal models (Fig. 4). One respondent (from a European NRA) chose the ‘Other’ option and indicated that compliance with a legislative requirement to apply the 3Rs was the most important factor.

Despite all 13 responses indicating that use of NATs was important, two respondents provided information related to their perception of barriers to adoption of NATs. Both responses indicated the view that there is insufficient evidence that NATs add value over current animal-based approaches. One of the two respondents also indicated that lack of in-house expertise with NATs was a barrier. The other respondent commented that animal tests are mandated in many pharmacopeia and that this prevents adoption of NATs.

**Fig. 5:**
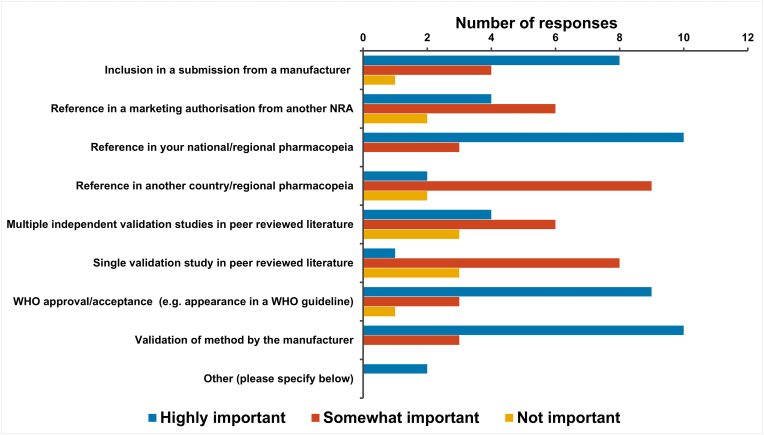
Factors that influence an NRAs decision to adopt NATs in quality control and batch release testing.

The survey went on to ask what factors influenced their NRA to adopt NATs for quality control and batch release testing of biological therapeutics. The survey allowed respondents to select multiple factors and to rank each as highly important, somewhat important and not important (Fig. 5).

All 13 NRAs answered the question. The most important factors (rated as highly important) were: inclusion of a specific NAT in their national/regional pharmacopeia (77 %; 10/13), validation of the NAT by a manufacturer (77 %; 10/13), WHO acceptance/approval of the NAT (69 %; 9/13) and inclusion of the NAT in a submission from a manufacturer (62 %; 8/13). Respondents indicated that the following factors were somewhat important: Reference of the NAT in other national pharmacopeia (69 %; 9/13) and a single validation study in peer reviewed literature (62 %; 8/13).

**Fig. 6:**
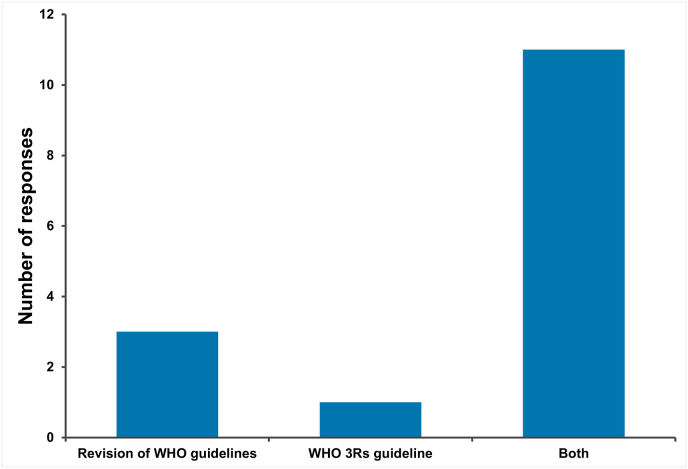
Value from an NRA perspective of proposed changes to WHO guidance with respect to adoption of 3Rs practices in quality control and batch release testing.

Respondents of the NRA part of the survey were also asked if revisions to WHO guidelines for biological therapeutics and vaccines, and/or a general guideline on applying the 3Rs, would influence the adoption of 3Rs practices for quality control and batch release testing requirements within their country (Fig. 6). An overwhelming majority 85 %; 11/13) indicated that both revision of WHO guidelines and a general 3Rs guideline would be beneficial to the adoption of NATs.

## 5 NCL responses

### 5.1 Understanding NCLs current use of NATs

**Fig. 7:**
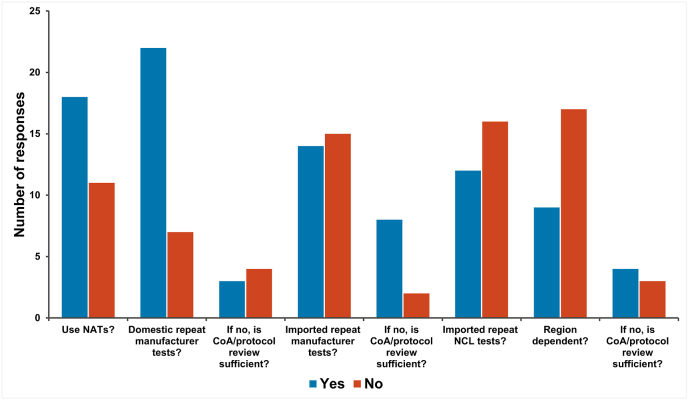
Understanding how NCLs currently use animal-based testing methods and NATs for quality control, batch release testing.

Questions were asked to better understand the current landscape with respect to the use of animal-based testing methods and NATs for quality control, batch release testing (Fig. 7). Most respondents (62 %; 18/29) indicated that NATs are used by their NCL.

Most respondents (75 %; 22/29) indicated that, for products manufactured in their country, they repeat quality control (QC) tests that have already been performed by the manufacturer. Of those that said that they did not repeat tests performed by manufacturers, two indicated that this was because they accepted a certificate of analysis and/or a protocol review as sufficient justification to waive the additional testing. The situation was somewhat different for imported products where there was an even split between respondents for NCLs who are (52 %; 15/29) and are not (48 %; 14/29) required to repeat quality control, batch release testing performed by the manufacturer. Of those that do not repeat these tests, most indicated that they accept a certificate of analysis (CoA) and/or a protocol review as sufficient justification to waive the additional testing. Where products are imported having been already released or tested by another NCL, a modest majority (57 %; 16/28) indicated that they would not repeat QC testing and most respondents (65 %; 17/26) indicated that the decision to repeat QC testing was not dependent on the region from which the NCL test data was received. Of those that indicated that they didn't repeat QC testing, a modest majority (57 %; 4/7) indicated that a certificate of analysis and/or protocol review or batch release certificate from the NCL of the country/region of origin was sufficient justification to waive the additional testing.

Most NCLs (62 %; 18/29) that responded to the survey indicated that they shared quality control and batch release testing responsibilities and/or results/certificates with other NCLs or networks. Some respondents (24 %; 7/29) indicated that their NCL performed animal-based testing for the WHO pre-qualification programme [7] and two of these NCLs indicated that they would use a NAT for the same product for release to a local market.

### 5.2 3Rs awareness and practice

**Fig. 8:**
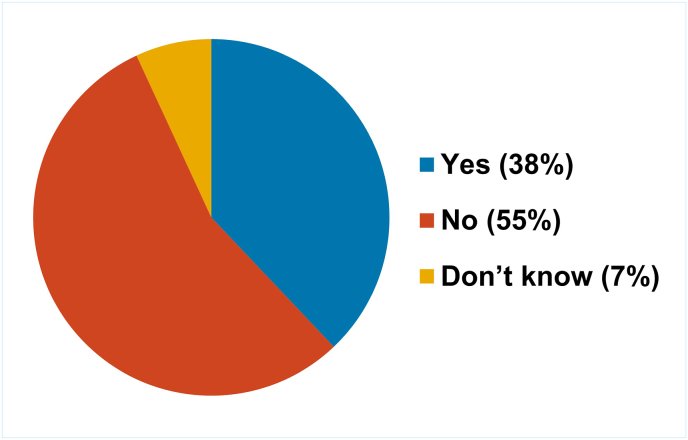
Proportion of NCLs that have a specific 3Rs policy.

Similar to the situation reported by the NRA respondents to the survey, most NCLs (16/29) that responded to the survey did not have a dedicated 3Rs policy (Fig. 8).

**Fig. 9:**
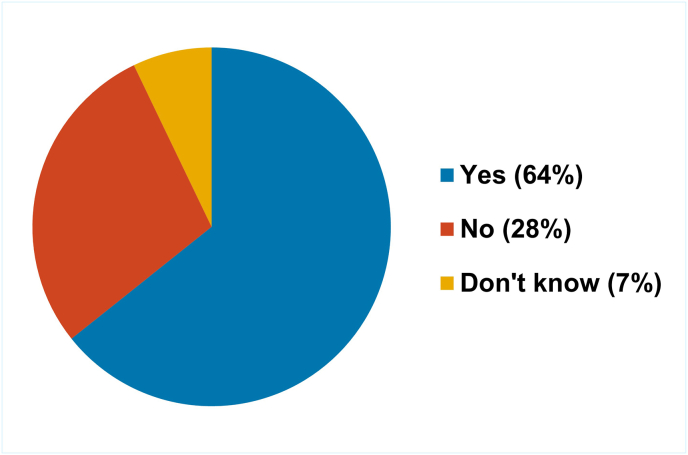
Proportion of NCLs that align themselves with national or international policy with respect to the 3Rs.

However, most respondents (18/29) indicated that their NCL did align itself with national or international 3Rs policy (Fig. 9) for example, most European NCLs would be aligned to European Union Directive 2010/63 EU on the protection of animals used for scientific purposes.

**Fig. 10:**
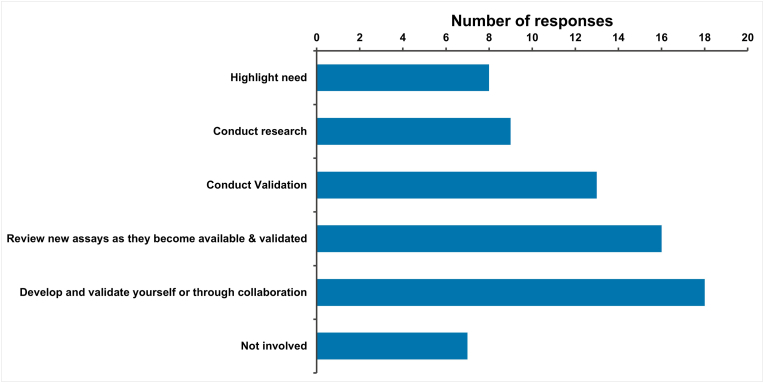
Understanding how NCLs are currently involved in the development of NATs for quality control, batch release testing.

Respondents were asked how involved their NCL was in the development of NATs (Fig. 10). Several options were offered in the survey and multiple selections were allowed (a total of 66 responses were given). Seven NCLs were not involved in NAT development. From the remaining respondents, the most common response (25 %; 18/71) was that they were actively involved in the development or validation of NATs either independently or via collaboration. The next most common response (23 %; 16/71) was from NCLs who review NATs as they become available and validated. Some NCLs conduct NAT validation (18 %; 13/71) or research (13 %; 9/71) and others (11 %; 8/71) highlight the need for NAT development.

**Fig. 11:**
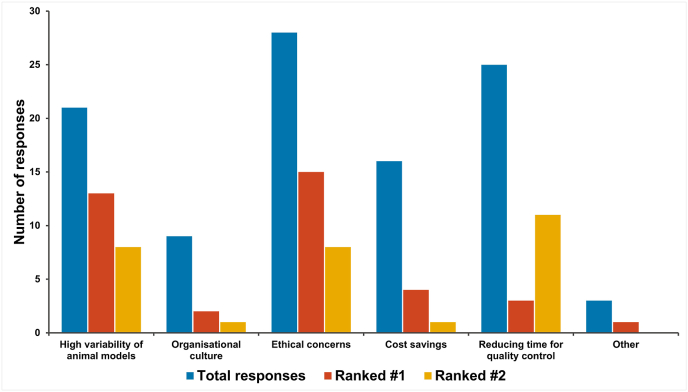
Reasons why NCLs regard adoption of NATs in quality control and batch release testing to be important.

NCLs were asked whether it is important that the 3Rs are applied to quality control, batch release testing of biological therapeutics as the NRAs. Similarly to the NRA data, all 27 respondents that answered the question state that they thought the 3Rs were important in this regard. When asked why (respondents were able to choose multiple options and to rank the top two responses; Fig. 11), respondents indicated that the most important factor was ethical concern for animal welfare, followed by reducing time for QC testing, high variability of data from animal models and cost savings. One respondent (from a European NCL) chose ‘Other’ as their most important factor and indicated that, in situations where an *in vitro* test method results in a superior control strategy for a product, the principal reason for selecting that method is that it is better from a “human ethical perspective” because the it optimises GC control of the product.

Five NCL respondents also provided information around the barriers to adoption of NATs. All suggested that a lack of in-house expertise with NATs was a barrier to adoption and, echoing the views of the NRA respondents, four NCLs also indicated that that there is insufficient evidence that NATs add value over current animal-based approaches.

**Fig. 12:**
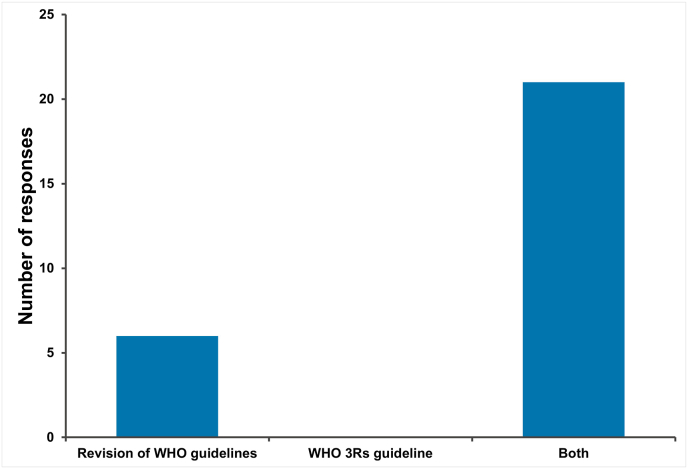
Value from an NCL perspective of proposed changes to WHO guidance with respect to adoption of 3Rs practices in quality control and batch release testing.

As with the NRA part of the survey, NCL respondents were also asked if revisions to WHO guidelines for biological therapeutics and vaccines, and/or a general guideline on applying the 3Rs, would influence the adoption of 3Rs practices for quality control, batch release testing requirements within their country (Fig. 12). An overwhelming majority indicated that both revision of WHO guidelines and a general 3Rs guideline would be beneficial to the adoption of NATs.

## 6 Discussion

Historically, confirmation of batch-to-batch safety and efficacy, and monitoring of production consistency for vaccines and biological therapeutics has involved testing in animals. More recently, better quality control measures (such as in-process controls, an understanding of critical quality attributes, and implementation of consistency approaches in manufacturing) and NATs have been increasingly used resulting in reduction or elimination of routine animal test methods [[8], [9], [10], [11], [12]]. It is widely acknowledged that for quality control and batch release purposes, NATs are considered superior to animal tests due to improved specificity and precision. These NATs have the additional advantage of reducing costs and duration of testing [13,14]. In addition to the robust scientific argument to transition away from animal testing paradigms there are also ethical and animal welfare concerns as the methods themselves can cause significant pain and distress to the animals [[15], [16], [17], [18]].

This survey set out to gain greater understanding around how animals are used in the regulation of quality control and batch release testing of biological products and to explore barriers and opportunities for greater implementation of the 3Rs [1]. It is the second of a pair of surveys and follows on from the survey of manufacturers that was published in November 2022 [2].

This survey had two separate sets of questions for respondents from NRAs and NCLs and it was possible for a single respondent to fill out both sets of questions if they worked for an organisation that performed both functions.

There were only 13 responses from NRAs. The skew towards a greater response rate from NCLs (29 responses) may be in part because the survey was strongly supported by the WHO NCL network and actively distributed and promoted to this group. It is difficult to draw strong conclusions from the few NRA data but there were some interesting trends observed from the NRA responses. Although all 13 NRA respondents indicated that it is important that NATs (where available and scientifically appropriate) are applied to quality control and batch release testing of biological therapeutics, most (62 %; 8/13) indicated that they had not discussed the use of NATs with manufacturers for this purpose. Most (62 %; 8/13) also indicated that their country had regulations in place that required animal testing for QC and batch release testing. This raises questions about the regulatory landscape and how this may influence the uptake of NATs and the move away from animal-based testing methods. This includes how individual NRAs work with manufacturers on adoption of NATs, the nature and timing of these discussions and how both perceive the outcomes.

The ATT/GST test is still widely performed despite the fact that the WHO [4] and several national pharmacopeias [4,5,19,20] have deleted the requirement for this test from their guidelines. We specifically asked whether respondents were aware that the WHO had deleted the ATT/GST and 85 % (11/13) indicated that they were aware of the decision of ECBS in 2018. Sixty nine percent of NRA respondents indicated that they continue to receive ATT/GST data despite almost all not requesting this. This suggests that manufacturers are performing the test even when it is not required by the regulator. This contrasts with the data from the manufacturers survey [2] which suggested that manufacturers were performing the test because some NRAs still asked for it. The relatively low number of NRA responses to the survey may go some way towards explaining this disparity. Additional potential reasons for the continued use of the ATT/GST are that even though WHO no longer requires the ATT/GST (as approved by ECBS [6]), many individual WHO guidelines have yet to be updated to remove the test. When a regulatory authority no longer requires the ATT, the individual product licence generally has to be updated via a post approval change [21] to remove the test. From a global perspective this is not a trivial or harmonised process and can cause significant delays in the release of products.

It is encouraging that most NCLs that responded to the survey indicated that they have implemented the use of NATs for quality control, batch release testing. It is also encouraging to note that most respondents also indicated that they shared testing responsibilities and or data with others NCLs, hopefully reducing the need to repeat tests in multiple countries. The survey invited respondents to provide additional information about the specific NATs that have been implemented. Fifteen respondents provided more information with the majority (9/15) indicating that they had replaced the rabbit pyrogen test with the monocyte activation test or with a bacterial endotoxin test. Other NATs that were specifically mentioned were *in vitro* tests for Hepatitis B, Hepatitis A and inactivated polio vaccines. One of the key tasks of NCLs is to perform impartial assessments of vaccine batches to their safety and efficacy prior to marketing. It is not surprising that most NCLs indicated that they repeat tests that have been performed by domestic manufacturers. This is in line with WHO expectations: the NCL of the producing country is considered responsible for batch release testing of produced vaccines. When products are imported the picture is less clear, with half of respondents indicating that they repeated tests while the other half did not. Imported products may have been tested by the NCL of the producing country, and this may be sufficient for the importing country.

As well as asking about current approaches for quality control and batch release testing, the survey for both NRAs and NCLs also sought to understand how both organisations viewed opportunities and barriers for adoption of the 3Rs. For both sets of respondents there was a mixed response when asked if their organisation had a dedicated 3Rs policy with half (NRA) or less than half (NCL) indicating that they had such a policy. Interestingly, NCL respondents indicated that their organisation aligned with national policy on the application of the 3Rs. Most of these NCLs were in Europe and would be aligned with EU directive 2010/63 EU on the protection of animals used for scientific purposes. There were several non-EU NCLs that aligned to national policies on the 3Rs, these were from North America, South America and Asia. This emphasises the wide reach and embedded nature of the 3Rs globally. When asked about the threshold of information required by NRAs to adopt NATs it was interesting to note that this was dependent on adoption in national or international regulatory guidance e.g. pharmacopeia or WHO guidance. This highlights the importance for these guidance documents to be regularly updated to ensure that the latest developments in NATs are included. It is encouraging to note that most NRAs also indicated that they would value data from NATs in submissions from manufacturers that included validated NAT methods. It would be beneficial if there was a coordinated effort between manufacturers, NRAs and those responsible for drafting national pharmacopeias and WHO guidance to ensure that validated NATs are included when these documents are revised.

Both sets of respondents indicated that, when available and scientifically justified, NATs should be used for quality control, batch release testing. Ethical reasons were cited as most important for this followed by the potential to reduce the time for quality control testing and the fact that *in vivo* test methods are highly variable when compared to *in vitro* approaches [22,23]. One clear difference between the responses from NRAs and NCLs was that respondents from NCLs rated cost savings as an important factor for the transition to using NATs, which is not surprising as NCLs perform tests themselves whereas NRAs do not. Given that ethical arguments for and against animal use for scientific purposes vary from region to region, more work may need to be done to demonstrate the scientific advantages of NATs and the 3Rs.

Despite all respondents from both surveys indicating that NATs were important it is clear that barriers to their adoption still exist. Seven respondents (two NRAs and five NCLs) provided information related to this. Two main reasons were given; firstly, that there was lack of local expertise with NATs and secondly, that there was insufficient evidence that NATs offer added value over current animal-based testing methods. This raises some questions around how these respondents regard the value of NATs: What is missing from the current NAT literature that causes this lack of confidence? Is this related to specific NATs, for specific products/test types only? How did the respondents interpret ‘insufficient evidence of added value’? It would be valuable to follow-up on this in future stakeholder engagement activities.

This survey was one of several stakeholder engagement activities (including a separate survey to biological therapeutic manufacturers [2] and a number of regional stakeholder workshops) [24] conducted as part of a project to review animal testing requirements within WHO guidelines [1]. All relevant, publicly accessible WHO guidance documents [25] for the quality, safety and efficacy of vaccines and biological therapeutics have been reviewed, and recommendations will be made to WHO ECBS in October 2023 to update these guidelines to promote more harmonised adoption of 3Rs principles in biological therapeutics batch release testing. It is encouraging that most respondents indicated that such revisions would help them to apply 3Rs approaches in the future. There was very strong support for WHO to produce a general guideline on applying the 3Rs to quality control, batch release testing of vaccines and biological therapeutics and this will form part of the recommendations in the final report to WHO ECBS.

In conclusion, it is clear that the regulatory community support the use of NATs and the 3Rs in quality control batch release testing of vaccines and biological therapeutics. It is also clear that there is a great deal of work to do to enable this to happen. Whilst there is widespread acceptance that NATs are superior to animal testing paradigms, the transition from the latter to the former remains challenging. Even when an animal test lacks scientific value, for example the ATT, the test is still requested, performed and data is submitted to regulatory authorities. There is a clear case for a harmonised approach to facilitate adoption of NATs and all key stakeholders need to be aligned in how to achieve this. The NC3Rs and the working group facilitating this project are optimistic that revision of WHO guidelines and the publication of a general 3Rs guidance document will contribute to achieving this goal.

## Appendix 1

The survey contained the following questions:

* indicates questions where a response was optional

### Section 1. Demographic Information

1a. Name of principle contact(s) completing the survey*
1b. Organisation
1c. Where (which country) is your facility located?
1d. Email address of individual(s) completing the survey*
1e. Are the NCL and NRA the same, or different/separate organisations in your country?
1f. Are you responding from the perspective of an NRA an NCL, or both?

### NRA Section 2. Quality control and batch release testing practices

2a. Does your country share regulatory decisions and quality control/batch release responsibilities with other countries within your region (e.g. Europe)?
2b. Has your organisation had discussions with a manufacturer regarding post-approval changes for the adoption of non-animal methods (replacement of a current method) for use in the quality control and/or batch release testing of their product?

If yes, were these discussions at the pre-approval or post-approval stage (or both)?

2c. Are your national/regional guidance documents in alignment with the WHO guidelines?
2d. Are there regulations in place within your country that would require a manufacturer to perform animal testing for quality control/batch release testing of their product?
2e. Regarding the innocuity test (also referred to as the abnormal toxicity test, general safety test or undue toxicity test), please indicate the following:
  ⁃ Are you aware that the WHO no longer requires the innocuity test?
  ⁃ Does your organisation still receive submissions with innocuity test data?
  ⁃ Does your organisation still request innocuity test data in specific circumstances? - (please provide details below)

### NRA Section 3. Opportunities and barriers to the 3Rs

3a. Does your NRA have a 3Rs policy or statement on the use of animals in National Control Laboratory batch release testing of biologicals?

If yes, please provide a link to the policy if possible:

3b. Please indicate the threshold of information you, as a regulator require to adopt a non-animal alternative for quality control and batch release testing of biologicals. (Select all that apply)
  ⁃ Inclusion in a submission from a manufacturer
  ⁃ Reference in a marketing authorisation from another NRA
  ⁃ Reference in your national/regional pharmacopeia
  ⁃ Reference in another country/regional pharmacopeia
  ⁃ Multiple independent validation studies in peer reviewed literature
  ⁃ Single validation study in peer reviewed literature
  ⁃ WHO approval/acceptance (e.g. appearance in a WHO guideline)
  ⁃ Validation of method by the manufacturer
  ⁃ Other (please specify below)
3c. Would revisions to the WHO guidelines for biologics and vaccines, and/or a general guideline on applying the 3Rs, influence the adoption of 3Rs practices for lot release testing requirements within your country?
3d. Is it important to your organisation that non-animal alternatives (where available and scientifically appropriate) are applied to quality control and batch release testing of biologics?
3e. If yes, why is this important? Choose all that apply and indicate the two most important drivers:
  ⁃ High variability of data/outcomes from use of animal models in batch release testing
  ⁃ Organisational culture
  ⁃ Ethical concerns for animal welfare
  ⁃ Cost savings
  ⁃ Reducing time for product quality control testing
  ⁃ Other (please specify)
3f. If no, what are the barriers to prevent this? Choose all that apply and indicate the two most important barriers:
  ⁃ Lack of in-house expertise in non-animal approaches
  ⁃ Non-animal approaches are too expensive
  ⁃ Insufficient evidence of added value over animal test method
  ⁃ Current non-animal models are not fit for purpose
  ⁃ Other (please specify)
3g. If you have any additional comments you wish to make regarding the 3Rs and biologics within your organisation, please include them below

### NCL Section 2. Quality control and batch release testing practices

2a. Does your organisation share quality control/batch release testing responsibilities and/or results/certificates with any other NCL or network?
2b. Does your organisation perform any animal testing for the WHO prequalification programme?

If yes, would your organisation use a non-animal test method for your local market for the same product?

2c. Have non-animal alternatives to animal tests for quality control or lot release been introduced into your NCL?
2d. For products manufactured in your country, are repeat quality control/batch release tests done by the NCL which have already been performed by the manufacturer?

If no, is only certificate of analysis and/or protocol review sufficient?

2e. For products imported into your country, does the NCL repeat quality control/batch release testing in animals that have already been performed by a manufacturer?

If no, is the certificate of analysis and/or protocol review sufficient?

2f. For products imported into your country, are you required to repeat quality control/batch release testing in animals that have already been released or tested by another NCL?

Is this dependent on the region/country where the product has already been tested?

If no, is only certificate of analysis and/or protocol review sufficient or batch release certificate from NCL of country/region of origin?

3a. Does your NCL have a 3Rs policy or statement on the use of animals in National Control Laboratory batch release testing of biologicals?

If yes, please provide a link to the policy if possible:

3b. Does your NCL align itself with national or international policies regarding the 3Rs?

If yes, please list the policies here:

3c. How involved is your organisation in the development of non-animal alternatives for quality control and batch release testing of biologics? Select all that apply
  ⁃ Highlight need (e.g. by publication)
  ⁃ Conduct research
  ⁃ Conduct validation
  ⁃ Review new assays as they become available and validated
  ⁃ Develop and validate yourself or through collaboration
  ⁃ Not involved
3d. Would revisions to the WHO guidelines for biologics and vaccines, and/or a general guideline on applying the 3Rs, influence the adoption of 3Rs practices for lot release testing requirements within your country?
3e. Do you think it is important that 3Rs approaches (where available and scientifically appropriate) should be applied to quality control and batch release testing of biologicals?
3f. If yes, what are the reasons you think this is important? Choose all that apply and indicate the two most important drivers:
  ⁃ High variability of data/outcomes from use of animal models in batch release testing
  ⁃ Organisational culture
  ⁃ Ethical concerns for animal welfare
  ⁃ Cost savings
  ⁃ Reducing time for product quality control testing
  ⁃ Other (please specify)
3g. If you think not, what are the barriers to prevent this? Choose all that apply and indicate the two most important barriers:
  ⁃ Lack of in-house expertise in non-animal approaches
  ⁃ Non-animal approaches are too expensive
  ⁃ Insufficient evidence of added value over animal test method
  ⁃ Current non-animal models are not fit for purpose
  ⁃ Other (please specify)
3h. If you have any additional comments you wish to make regarding the 3Rs and biologics within your organisation, please include them below.
